# A Bibliometric Analysis of 8,276 Publications During the Past 25 Years on Cholangiocarcinoma by Machine Learning

**DOI:** 10.3389/fonc.2021.687904

**Published:** 2021-09-07

**Authors:** Zeyu Zhang, Zhiming Wang, Yun Huang

**Affiliations:** ^1^Department of General Surgery, Xiangya Hospital, Central South University, Changsha, China; ^2^Department of Hepatobiliary Surgery, Xiangya Hospital, Central South University, Changsha, China

**Keywords:** cholangiocarcinoma, bibliometrics, machine learning, natural language processing**, latent Dirichlet allocation**

## Abstract

**Introduction:**

Cholangiocarcinoma (CCA) is the second most common hepatic malignancy. Progress and developments have also been made in the field of CCA management along with increasing scientific publications during the past decades, which reflect topics of general interest and suggest the future direction of studies. The purpose of this bibliometric study is to summarize scientific publications during the past 25 years in the field of CCA using a machine learning method.

**Material and Methods:**

Scientific publications focusing on CCA from 1995 to 2019 were searched in PubMed using the MeSH term “cholangiocarcinoma.” Full associated data were downloaded in the format of PubMed and extracted in the R platform. Latent Dirichlet allocation (LDA) was adopted to identify the research topics from the abstract of each publication using Python.

**Results:**

A total of 8,276 publications related to CCA from the last 25 years were found and included in this study. The most type of publications remained little changed, while the proportion of clinical trials remained relatively low (7.24% as the highest) and, more significantly, with a further downward trend during the recent years (1.42% in 2019). Neoplasm staging, hepatectomy, and survival rate were the most concerning terms among those who are diagnosis-related, treatment-related, and prognosis-related. The LDA analyses showed chemotherapy, hepatectomy, and stent as the highly concerned research topics of CCA treatment. Meanwhile, conversions from basic studies to clinical therapies were suggested by a poor connection between clusters of treatment management and basic research.

**Conclusion:**

The number of publications of CCA has increased rapidly during the past 25 years. Survival analysis, differential diagnosis, and microRNA expression are the most concerned topics in CCA studies. Besides, there is an urgent need for high-quality clinical trials and conversions from basic studies to clinical therapies.

## Introduction

Cholangiocarcinoma (CCA), as the second most common hepatic malignancy, comprises about 15% of liver tumors and 3% of gastrointestinal tumors (1, 2). CCA includes multiple malignancies of the biliary system: intrahepatic, perihilar, and distal types, according to the primary site of the tumor (3). CCA is a relatively rare malignancy with a very poor patient prognosis; however, its incidence has continuously increased globally during these years, causing a significant health problem worldwide (4, 5). In the meantime, progresses and developments have also been made in the field of CCA management along with increasing scientific publications during the past decades, which reflects topics of general interest and suggests the future direction of studies. Thus, bibliometric analyses are generally needed to make clear these progresses and developments by investigating published literature. However, as far as we know, no such bibliometric study has been introduced in the field of CCA.

Latent Dirichlet allocation (LDA), as an essential algorithm of natural language processing (NLP) which constitutes a group of machine learning methods to analyze human language, is most commonly utilized for scientific publication analyses. It can function as recognizing research topics and subsequently sorting publications into these topics (6, 7). The purpose of this study is to summarize the scientific publications of CCA in the past 25 years. Moreover, by comprehensively analyzing the research topics with machine learning, we are also managed to give insights into scientific developments and hot zones of CCA and, more importantly, reveal potential future research foci in the field of CCA.

## Materials and Methods

Given the possible publication index delay of literature in 2020, literature in English from 1995 to 2019 were searched and downloaded in PubMed using the MeSH term “cholangiocarcinoma.” An R package “Bibliometrix” was used for data extraction, including the publication year, publication type, MeSH terms, and abstract (8). Only MeSH terms with more than 20 times of appearance were enrolled in this analysis. Additionally, an ethical approval was waived because it was a bibliometric analysis.

To analyze in detail the research topics of an enormous number of scientific publications, LDA, as a machine learning method, was adopted to identify the research topics from the abstract of each publication in the Python platform. LDA would create a series of glossary of terms depending on the coexistence of vocabularies in the literature series. Subsequently, the two most suitable research topics of each literature were calculated, according to the frequency of the appearance of these glossary vocabularies in each literature. Furthermore, cluster analyses with the Louvain algorithm were administrated to investigate the associations between identified topics.

R platform and Excel software were used for the visual illustrations, while the topic network was realized by Gephi software (9). All the used codes, including R platform and Python platform, were available on GitHub (https://github.com/yan-wen0614/Medicine-Bibliometric-Analysis).

## Results

**Figure 1:**
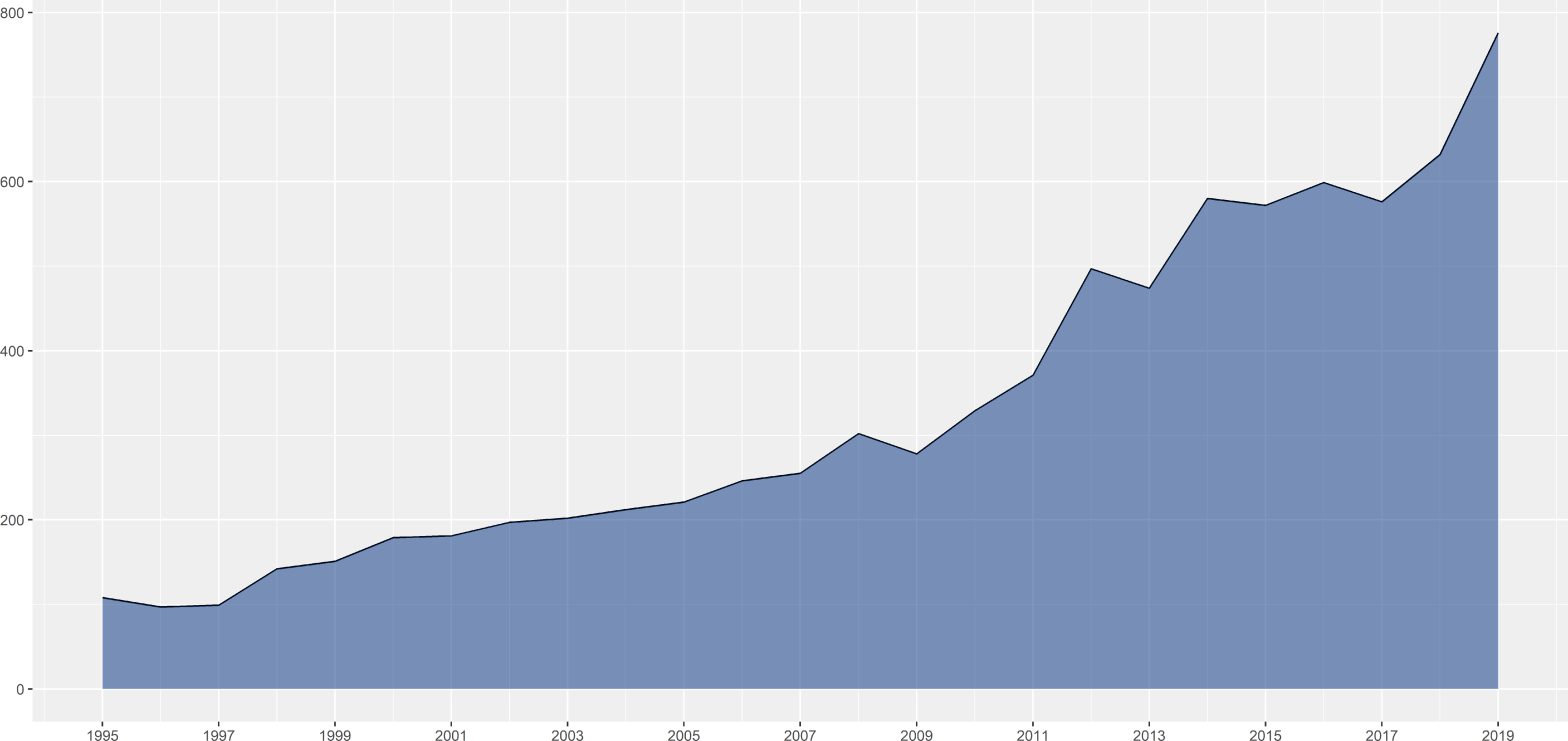
Scientific publications per year.

**Figure 2:**
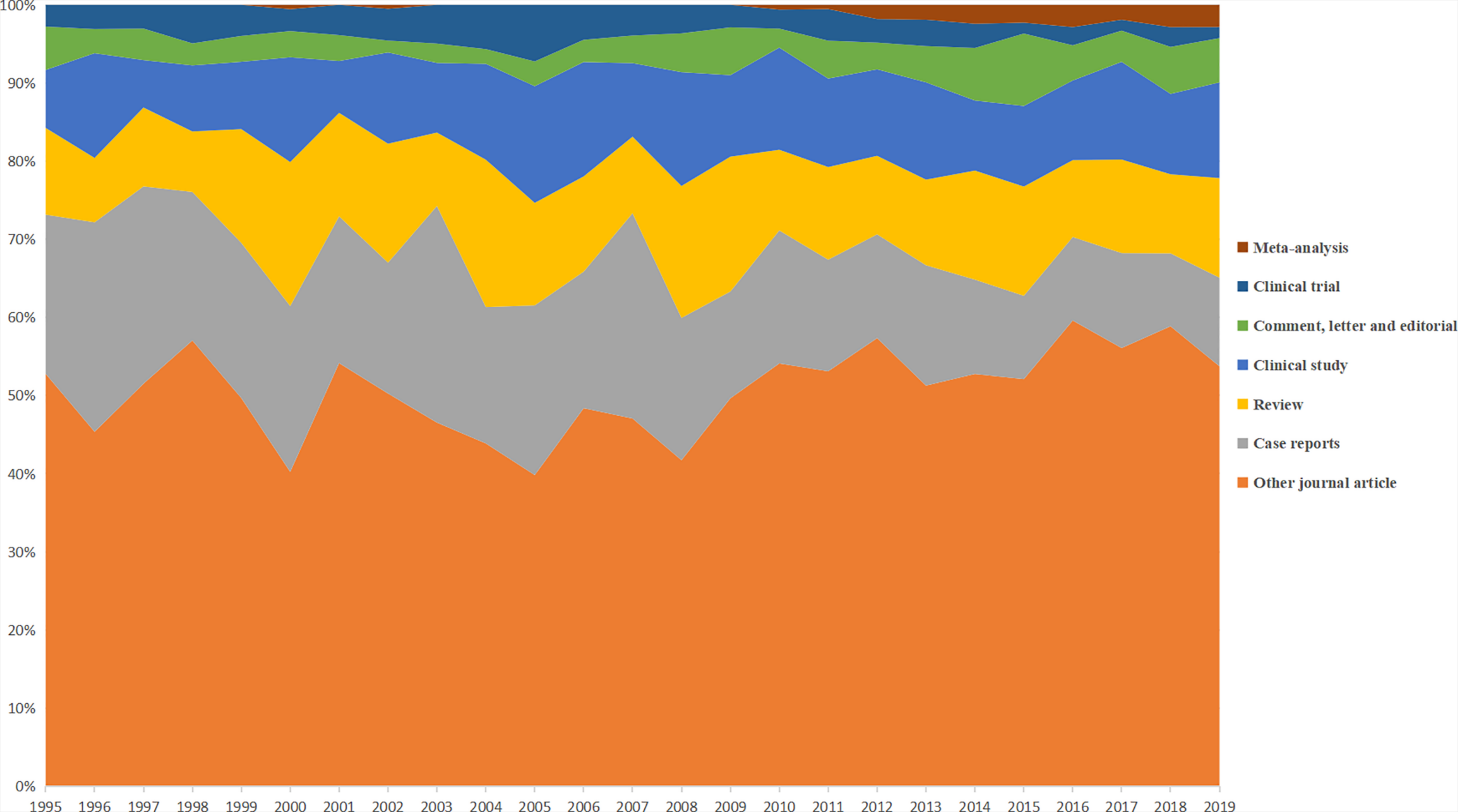
Distribution of publication types per year.

**Figure 3:**
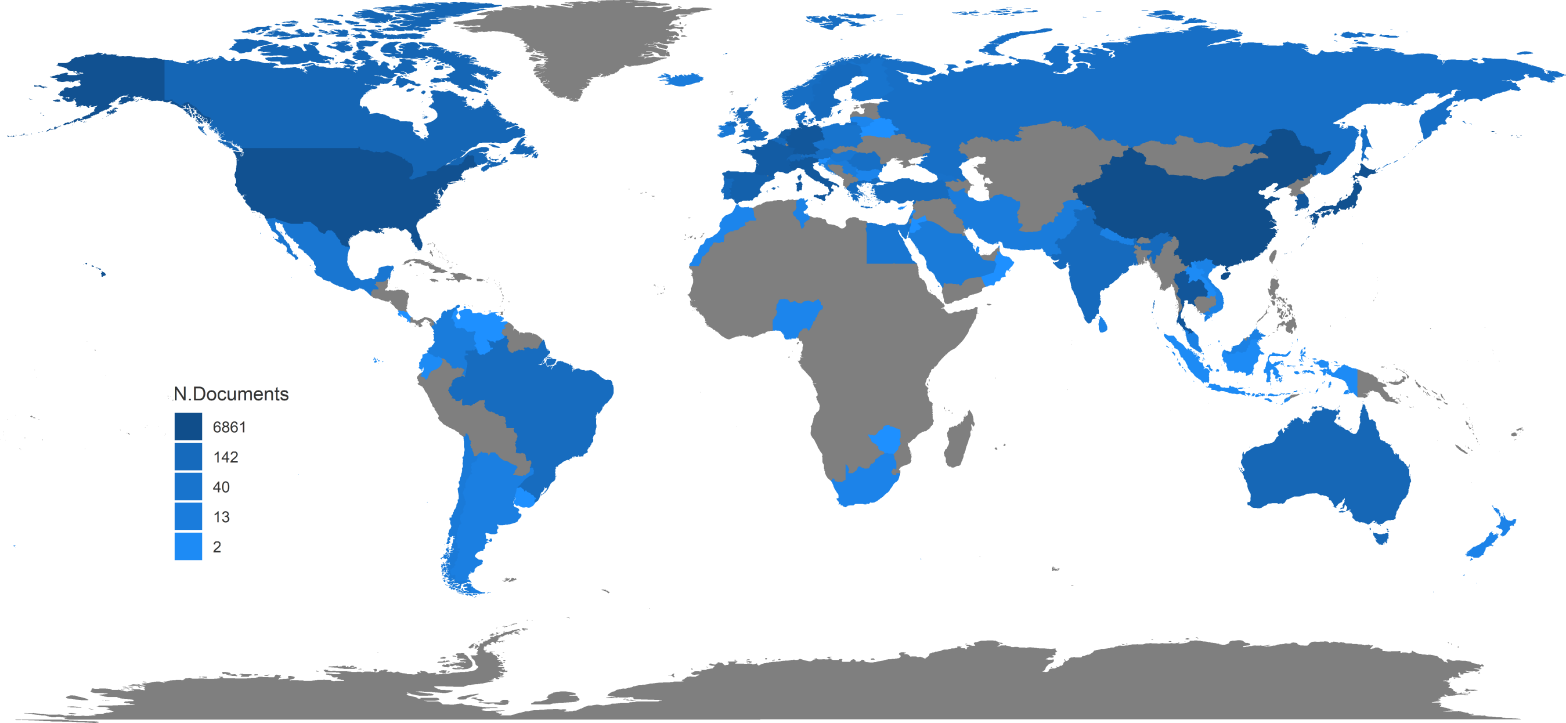
Country scientific production.

A total of 8,276 CCA-related publications from the past 25 years were finally searched and included in the bibliometric analyses. **Figure 1** shows that there was a significant and constant growth of the annual CCA-related publications during the past 25 years from 108 in 1995 to 776 in 2019. The type of publications was roughly divided, as shown in **Figure 2**. While most types of publications remained little changed, the proportion of clinical trials remained relatively low (7.24% as the highest) and, more significantly, with a further downward trend during the recent years (1.42% in 2019). On the other hand, there was a significant increase in the proportion of meta-analysis, which firstly emerged in 2000, indicating the development of evidence-based medicine. Moreover, **Figure 3** shows the country scientific production. China, Japan, USA, Thailand, and Germany were the top five countries producing CCA-related publications. In addition, the top 10 affiliations with the highest scientific production are listed in **Table 1**.

**Table 1:** Top 10 affiliations with highest scientific production.

Name of affiliation	Number of article
Khon Kaen University	1598
Fudan University	531
Mayo Clinic	436
University Of Ulsan College Of Medicine	367
Nagoya University Graduate School of Medicine	341
Second Military Medical University	291
Harvard Medical School	272
Kumamoto University	247
National Cancer Institute	236
Sun Yat-sen University	227

### MeSH Term Analyses

After excluding MeSH terms with less than 20 appearances, 632 terms with 86,853 times of appearances were focused. **Table 2** reveals some general terms of CCA studies. Notably, retrospective studies and intrahepatic CCA respectively took the most attention of researchers with a wide disparity compared with other terms.

**Table 2:** Top three terms in general issues of cholangiocarcinoma (CCA) studies during the past 25 years.

Category	Name of affiliation	Number of occurrence
Study subject	Human	8,090
	Animal	1,031
	Cell line	849
Age group	Middle aged	4,399
	Aged	3,913
	Adult	2,680
Study design	Retrospective studies	618
	Follow-up studies	247
	Prospective studies	313
Location of CCA	Bile ducts, intrahepatic	2,114
	Bile ducts, extrahepatic	121

**Figure 4:**
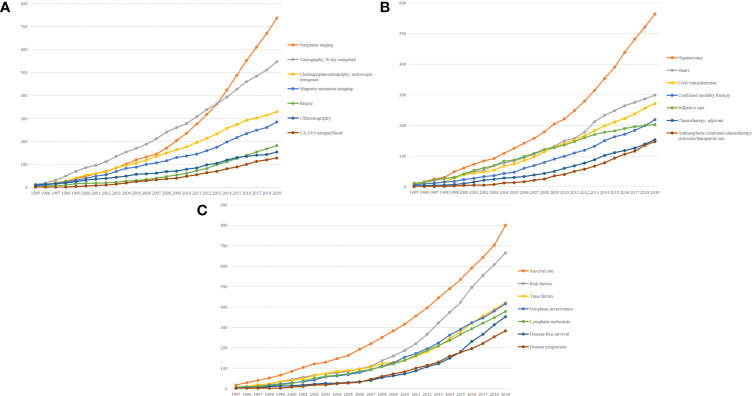
Top 7 MeSH terms concerning diagnosis **(A)**, treatment **(B)**, and prognosis **(C)** of cholangiocarcinoma and accumulative occurrences during the past 25 years.

We further divided clinical-related terms into diagnosis-related, treatment-related, and prognosis-related. As shown in **Figure 4**, neoplasm staging was the most concerning issue of diagnosis, which grew rapidly since 2007, followed by multiple examination methods. CA-19-9 was firstly mentioned in 1995; however, no further concern was addressed on CA-19-9 until 1999. Hepatectomy and liver transplantation were the leading terms in the treatment of CCA, for most of CCAs were intrahepatic. Stents, combined modality therapy, and adjuvant chemotherapy also received great attention. However, even with these comprehensive therapies, the high degree of concern on palliative care might indicate the poor prognosis of CCA patients. Among the prognosis-related terms, survival rate and risk factors were the leading terms. Compared with survival rate, disease-free survival, as a more comprehensive indicator of patient prognosis, gradually attracted the attention of researchers during the recent years.

### LDA Analyses

**Figure 5:**
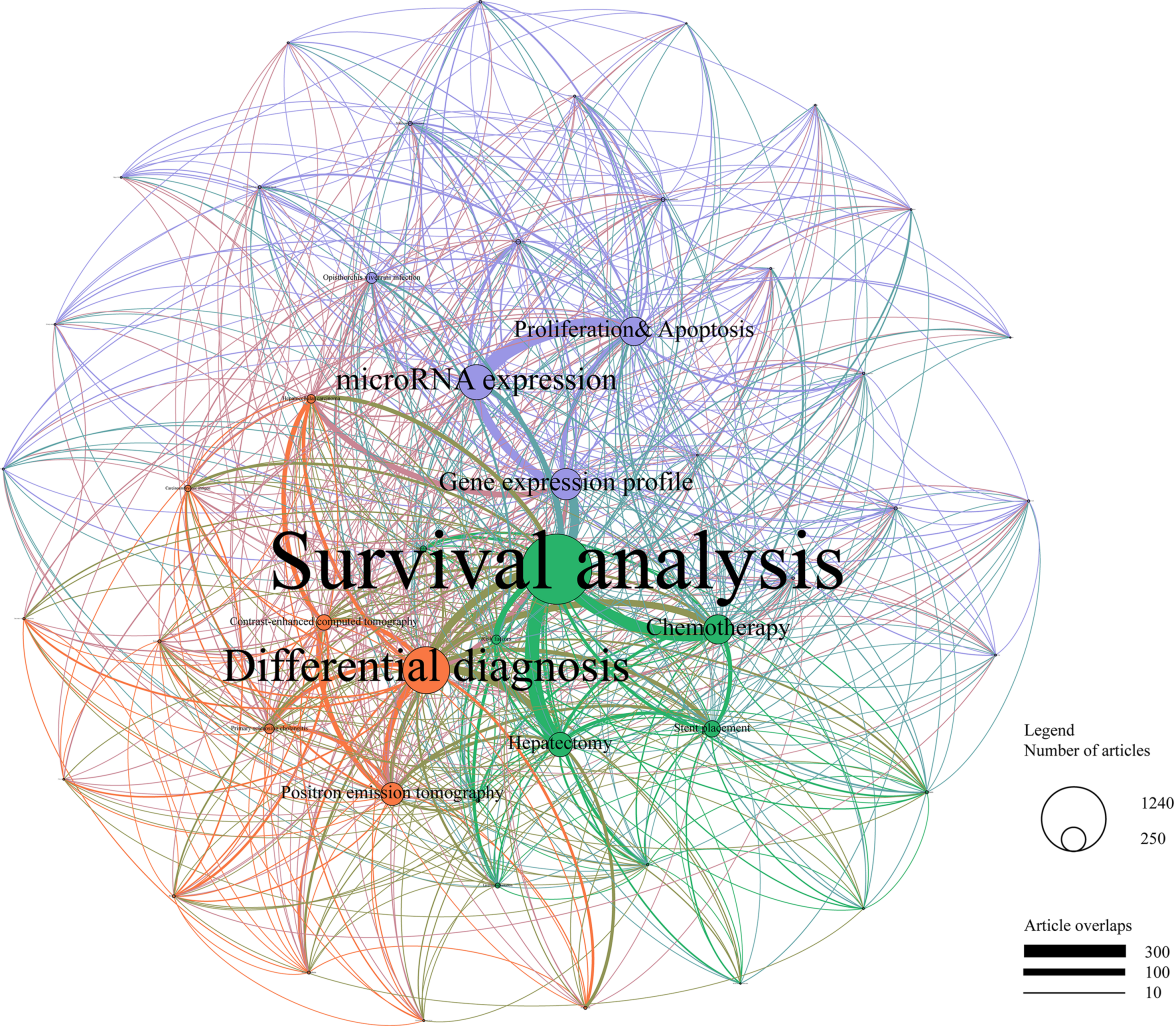
Topic cluster network by latent Dirichlet allocation. Green, treatment management; red, diagnosis research; purple, basic research. The size of the circle represents the number of papers in each topic, and the thickness of the line represents the weight of connection between each topic.

To further and comprehensively identify research foci of CCA, LDA analysis was applied among 7,321 publications with an abstract. Fifty research topics and an associated network were identified from these abstracts, as shown in **Figure 5**. The cluster analysis divided the network into three clusters as treatment management (in green), diagnosis research (in red), and basic research (in purple).

In the cluster of treatment management, survival analysis, chemotherapy, and hepatectomy were the top three research topics. Endeavors were also put on stent placement and risk factors. In the cluster of diagnosis research, differential diagnosis, positron emission tomography (PET), and contrast-enhanced computed tomography (CT) were the top three research topics. The cluster of basic research covered the largest proportion of these topics, with microRNA expression, gene expression profile, and proliferation & apoptosis as the top three focused topics in this cluster. Notably, the basic research cluster showed a poor connection with other clusters, while the strongest connections appeared between survival analysis and expression profiles of microRNA and gene, indicating that a large amount of basic researches failed to translate to clinical therapies.

## Discussion

This bibliometric study firstly adopted a machine learning method to analyze research foci of 8,276 CCA publications during the past 25 years. Over these years, the amount of CCA publications boosted expeditiously, with a significant increase of meta-analysis and a small proportion of clinical trials. LDA analyses showed that the most concerning research topics were survival analysis, differential diagnosis, and microRNA expression in the field of treatment management, diagnosis research, and basic research. Overall, this study suggested pressing requirements of not only clinical trials but also conversions from basic studies to clinical therapies.

According to the results of LDA analysis, basic research covered a large proportion of research topics. With the development of sequencing technology, gene and microRNA expression profiles emphasize the genomic complexity in occurrence and development of CCA. Multiple signaling pathways were also proved to play a role in CCA, including RTK signaling, AKT-mTOR signaling, FXR signaling, and Wnt signaling (10). The results also showed proliferation and apoptosis as the most concerned tumorigenic processes, rather than migration, invasion, or differentiation. These topics were strongly connected to survival analysis, however scarcely to diagnosis or clinical therapy, indicating they were often used for prediction and assessment of patient prognosis. However, they were relatively less studied with clinical diagnosis and therapy, suggesting more studies toward these issues.

Differential diagnosis was presented to be the most concerned in the cluster of diagnosis management with PET and CT as the most concerned imaging techniques. There is no specific radiology pattern of CCA, and enhancement patterns of hepatocellular carcinoma (HCC) can also present in CCA (11). Thus, misclassification between HCC and intrahepatic CCA should be prevented with priority, which was also shown in the LDA network. Generally, CT scan and magnetic resonance imaging (MRI) share the same significance in diagnosing CCA. Meanwhile, PET can be of special value in detecting lymph nodes and distant metastases (12). However, PET took a more significant role than MRI in CCA studies, which might be caused by the relatively advanced stage of CCA patients when diagnosed. Additionally, liquid biopsy has emerged with substantial attention using blood or urine for monitoring tumor recurrence, metastasis, or treatment responses in various types of cancer, including CCA. Compared with conventional biopsy, liquid biopsy is non-invasive, feasible, and cost-effective. Although this novel technique was not found in the present study due to the fact that it was reported on CCA mainly in 2020 and 2021, several studies have described the feasibility and effectiveness of liquid biopsy using circulating tumor DNA (13) or transcriptomic profile of extracellular vesicles (14) in managing CCA patients. However, a well-designed worldwide multicenter prospective study should be performed, and a unified standard of liquid biopsy is also needed. Based on these premises, liquid biopsy may greatly contribute to the precise treatment and a better patient prognosis among CCA patients.

Due to the highly aggressive biological behavior and the absence of symptoms in the early stage, the therapies were often compromised in patients diagnosed with CCA, which directly results in a poor patient prognosis. About only 25% of CCA patients are eligible for surgery, while chemotherapy is the first-line treatment for the rest (15). For patients who undergo surgery, adjuvant chemotherapy is also recommended to reduce the recurrence rate of CCA (16), which provides an explanation for the higher profile of chemotherapy than surgery. Jaundice is one of the main manifestations of CCA, calling for the need for biliary drainage. Stent, the main method for biliary drainage, can be used as a palliative treatment against jaundice or a preoperative intervention to ameliorate functions of future remnant liver (17). Compared with percutaneous drains, stent implantation provides a better quality of life without a drainage tube, making it important in treating CCA (18). Notably, even with many studies on carcinogenic mechanisms and signaling pathways, targeted therapies were not found in the research foci of the treatment management cluster, which might be caused by its early stage of development. Although targeted therapies of IDH1, IDH2, EGFR, and FGFR are under investigation with promising preliminary results (19–21), more therapies, other than chemotherapy, should be discovered from basic studies to cover a wider scope of CCA patients.

This study particularly highlighted tumor staging in the field of diagnosing CCA, and surgical interventions in treating CCA, which would also be the research foci within the next few years. Tumor staging can cover a large number of issues, from treatment selection to prognosis prediction. The AJCC staging system is currently most accepted and frequently used in CCA. While the established staging system, mainly consisting of patient and tumor characteristics, is constantly challenged and updated, researchers should be aware of the potential of novel biomarkers to be included in the future staging system. With the development of sequencing technologies, the expression pattern of particular gene sets is most likely to be included in the future staging system, regardless of tumor biopsy or liquid biopsy. Individualized treatment and precision medicine may be subsequently achieved. In terms of CCA treatment, surgical interventions provide the only cure for CCA patients, although a small proportion of patients are able to receive it. As novel surgical techniques and adjuvant therapies are created and developed, future efforts should be made to expand surgery indications or develop translational treatments to allow more CCA patients to receive curative surgery. Meanwhile, the development of new therapeutic targets can also contribute to a better patient prognosis, including targeted therapy, immunotherapy, and photodynamic therapy. Combinations of these treatments with surgery may be another breakthrough in treating CCA.

Limitations still existed in this study. Firstly, the MeSH term was used for searching publications and the PubMed database was subsequently chosen, while other databases were also available. Secondly, the abstract was used for LDA analysis, while we failed to use the full article. Lastly, the newest development with significance, such as immunotherapy, was not well discussed because of the design of this bibliometric study.

## Conclusion

The number of publications of CCA increases rapidly during the past 25 years. Survival analysis, differential diagnosis, and microRNA expression are the most concerned topics in CCA researches. Besides, there is an urgent need for high-quality clinical trials and conversions from basic studies to clinical therapies.

## Data Availability Statement

The original contributions presented in the study are included in the article/supplementary material. Further inquiries can be directed to the corresponding author.

## Author Contributions

All authors made substantive intellectual contributions to this study to qualify as authors. YH conceived of the design of the study. YH and ZW modified the design of the study. ZZ, ZW, and YH performed the study, collected the data, and contributed to the design of the study. ZZ and ZW analyzed the data. ZZ and ZW drafted the Result, Discussion, and Conclusion sections. YH drafted the Methods sections. ZZ, ZW, and YH edited the manuscript. All authors have agreed to be accountable for all aspects of the work in ensuring that questions related to the accuracy or integrity of any part of the work are appropriately investigated and resolved. All authors contributed to the article and approved the submitted version.

## Conflict of Interest

The authors declare that the research was conducted in the absence of any commercial or financial relationships that could be construed as a potential conflict of interest.

## Publisher’s Note

All claims expressed in this article are solely those of the authors and do not necessarily represent those of their affiliated organizations, or those of the publisher, the editors and the reviewers. Any product that may be evaluated in this article, or claim that may be made by its manufacturer, is not guaranteed or endorsed by the publisher.
